# Supplementary material for: End Sequence Analysis Toolkit (ESAT) expands the extractable information from single-cell RNA-seq data
Source: Genome Res. 2016 Oct;26(10):1397–410. doi: 10.1101/gr.207902.116 (PMC5052061; doi:10.1101/gr.207902.116)
Supplement: Supplemental Material [file supp_gr.207902.116_Supp_Materials.tar.gz › ESAT-esat_v0.1/doc/html/interfacebroad_1_1pda_1_1seq_1_1segmentation_1_1_read_filter_3_01_t_01_4.html]

ESAT: broad.pda.seq.segmentation.ReadFilter< T > Interface Reference


ESAT


- Main Page
- Related Pages
- Packages
- Classes
- Files

- Class List
- Class Index
- Class Hierarchy
- Class Members

All Classes Namespaces Files Functions Variables Enumerator Pages

- broad
- pda
- seq
- segmentation
- ReadFilter< T >

Public Member Functions |
List of all members

broad.pda.seq.segmentation.ReadFilter< T > Interface Reference

|  |  |
| --- | --- |
| Public Member Functions | |
| boolean | passes (T read) |
|  | |

## Member Function Documentation

|  |  |  |  |  |  |
| --- | --- | --- | --- | --- | --- |
| boolean broad.pda.seq.segmentation.ReadFilter< T >.passes | ( | T | *read* | ) |  |

Evaluates whether or not a given read alignment passes the filtering criteria. Filters can also modify the read itself by, for example setting orientation

Parameters
:   |  |  |
    | --- | --- |
    | read,the | read alignment |

Returns
:   true if it passes the filtering criteria.

---

The documentation for this interface was generated from the following file:

- java/broad/pda/seq/segmentation/ReadFilter.java


---

Generated on Fri Aug 15 2014 15:11:05 for ESAT by  

 1.8.7
